# Uterine collision tumor (PEComa and endometrioid carcinoma) in a tuberous sclerosis patient: a case report

**DOI:** 10.3389/fonc.2023.1244261

**Published:** 2023-08-09

**Authors:** Nektarios Koufopoulos, Ioannis S. Pateras, Christos Koratzanis, Alina-Roxani Gouloumis, Argyro-Ioanna Ieronimaki, Alexandros Fotiou, Ioannis G. Panayiotides, Nikolaos Vrachnis

**Affiliations:** ^1^ Second Department of Pathology, National and Kapodistrian University of Athens School of Medicine, “Attikon” University Hospital, Haidari, Greece; ^2^ Third Department of Obstetrics and Gynecology, National and Kapodistrian University of Athens School of Medicine, “Attikon” University Hospital, Haidari, Greece

**Keywords:** uterine PEComa, endometrioid carcinoma, collision tumor, tuberous sclerosis, case report

## Abstract

Perivascular epithelioid cell tumors are very rare mesenchymal neoplasms arising in various locations, such as the female genital tract, kidney, lung, prostate, bladder, pancreas, soft tissues, and bone. They possess a unique immunophenotype, co-expressing myogenic and melanocytic markers; molecular findings include mutations of tuberous sclerosis complex and translocations of transcription factor E3, a member of the microphthalmia transcription factor gene family. We herewith report a uterine collision tumor consisting of a perivascular epithelioid cell tumor and a moderately differentiated endometrial endometrioid carcinoma in a patient with genetically proven tuberous sclerosis; two leiomyomas were also found in contact with the tumor. Although two such cases one with a benign and another with a malignant perivascular epithelioid cell tumor have previously been reported, ours is, to our knowledge, the first reported in a tuberous sclerosis patient.

## Introduction

Perivascular epithelioid cell tumors (PEComas) are very rare mesenchymal tumors, including some previously reported entities, such as renal and hepatic angiomyolipomas, lymphangioleiomyomatosis, pulmonary and extrapulmonary clear cell (“sugar”) tumors, abdominopelvic sarcoma of perivascular epithelioid cells, clear cell myomelanocytic tumor of the falciform ligament/ligamentum teres, and other similar tumors in different sites (1–4). Most PEComas are sporadic, arising more frequently in women than men, and exhibit a wide age range (5). A subset of cases, less than 10%, are linked to the tuberous sclerosis (TS) complex, also known as Bourneville-Pringle disease (6). PEComas arise in several locations, including the kidney, lung, prostate, bladder, pancreas, soft tissues, and bone (7–9). The most common location in the female genital tract is the uterine corpus, the uterine cervix, vagina, vulva, broad ligament, retroperitoneum, and ovary being less frequently involved (7). Histologically, PEComas are composed of cells with epithelioid and/or spindle cell morphology, clear to granular cytoplasm, and a centrally located, round to oval or elongated nucleus. Those cells stain with both myogenic and melanocytic immunohistochemical markers (8).

Leiomyomas are the most common benign mesenchymal neoplasms, whereas endometrioid endometrial carcinoma is the most common type of corpus uteri carcinoma, accounting for approximately 80% of all endometrial carcinomas (10, 11).

Collision tumors are two or more separate primary tumors in the same anatomic location without histological admixture or the existence of an intermediate cell population zone. They may be benign or malignant, primary or secondary in any combination. In some cases, they are the first manifestation of an occult disease (12, 13).

We herewith describe a unique case of uterine PEComa and endometrioid carcinoma with the coexistence of uterine leiomyomas in a patient with the underlying genetic background of TSC and review the literature.

## Materials and methods

The surgical specimens underwent fixation in 10% buffered formalin. Formalin-fixed, paraffin-embedded blocks, were sectioned (4 μm thick) and the histological slides were stained with hematoxylin-eosin and immunohistochemical markers. The following antibodies were employed: anti-: CKAE1/AE3 (mouse monoclonal AE1/AE3 clone, Dako), ER (rabbit monoclonal EP1 clone, Dako), PR (mouse monoclonal PgR 636 clone, Dako), p16ink4a (mouse monoclonal JC2 clone, Zytomed), p53 (mouse monoclonal DO7 clone, Dako), MITF (mouse monoclonal C5/D5 clone, Cell Marque), HMB45 (mouse monoclonal HMB45 clone, Dako), Melan-A (mouse monoclonal A103 clone, Dako), SMA (mouse monoclonal 1A4 clone, Dako), desmin (mouse monoclonal D33 clone, Zytomed), calretinin (mouse monoclonal DAK-Calret 1 clone, Dako), h-caldesmon (mouse monoclonal h-CD clone, Dako), CD117 (rabbit polyclonal, Dako), S100 (rabbit polyclonal, Dako) MLH1 (mouse monoclonal ES05, DAKO), PMS2 (rabbit monoclonal EP51, DAKO), MSH2 (mouse monoclonal FE11, DAKO), and MSH6 (rabbit monoclonal EP49, DAKO) and β-catenin (rabbit monoclonal E247, Thermoscientific). Diaminobenzidine-chromogen substrate was used to visualize the staining and hematoxylin was used as counterstain. The patient has provided written informed consent. All procedures performed in the current study were approved by IRB (EΣ121/28-03-2023) in accordance with the 1964 Helsinki declaration, and its later amendments.

## Case report

**Figure 1:**
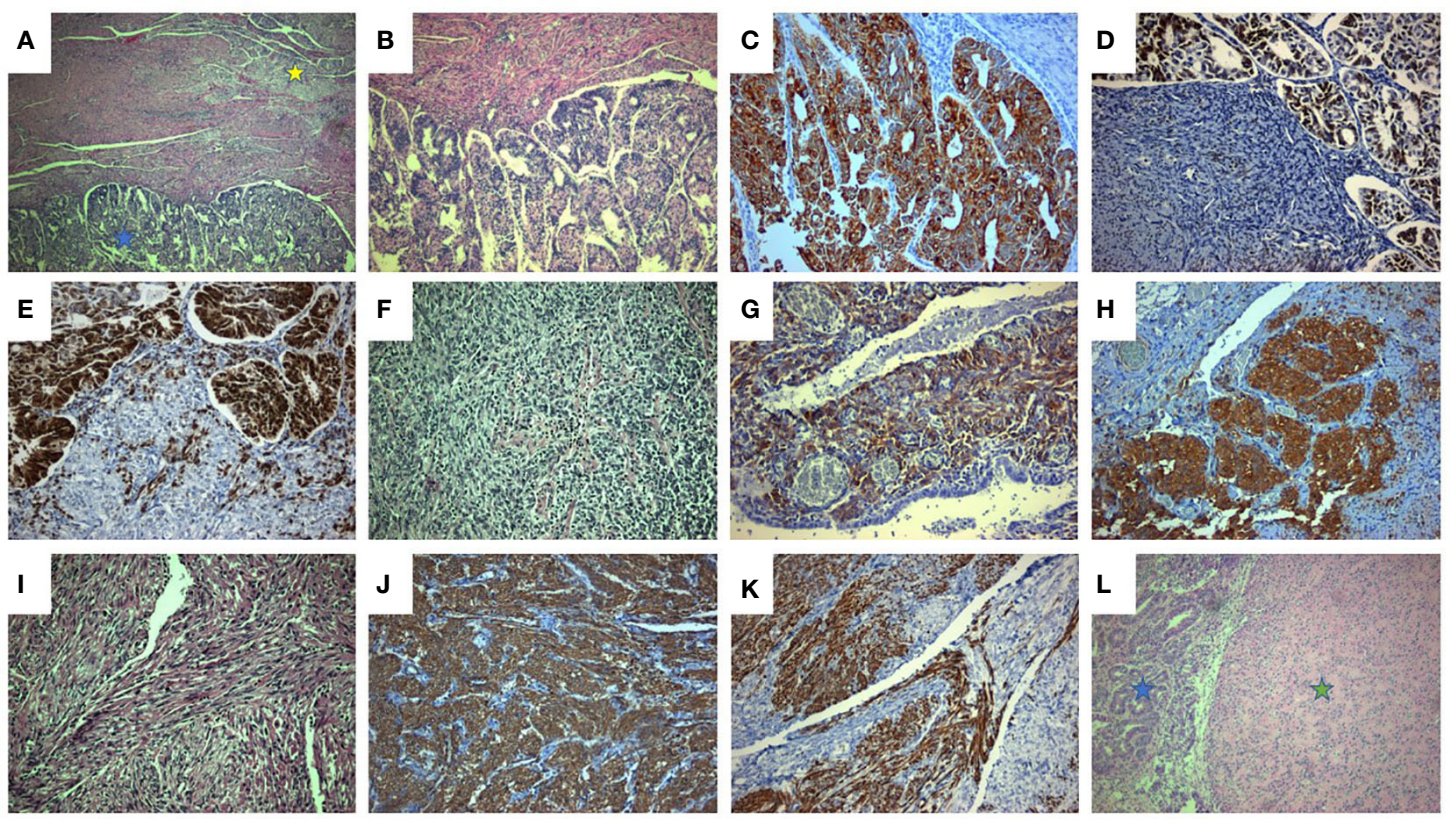
Coexistence of uterine endometrioid adenocarcinoma along with PEComa and leiomyoma. **(A)** Uterine endometrioid carcinoma (blue asterisk) associated with PEComa (yellow asterisk) (H&E x40). **(B)** The endometrioid carcinoma, displays mainly tubular and cribriform and in a lesser degree (estimated at 10%) solid architecture (solid architecture not shown). (H&E x 100). **(C-E)**. Endometrioid carcinoma: diffuse and intense CKAE1/AE3 (**C**, x200), ER (**D**, x200), and PR (**E**, x200) immunostaining. **(F-H)**. PEComa: epithelioid component (**F**, neoplastic cells with epithelioid morphology and clear cell cytoplasm, H&E x200) exhibiting strong HMB45 (**G**, x200) and MiTF (**H**, x200) immunostaining. **(I-K)**. PEComa: spindle cell component (I, neoplastic cells with spindle cell morphology and clear cell cytoplasm, H&E, x200) exhibiting strong Desmin (**J**, x200) and SMA (**K**, x200) immunostaining. **(L)**. Uterine leiomyoma (green asterisk) associated with endometrioid carcinoma (blue asterisk) (H&E, x100).

**Figure 2:**
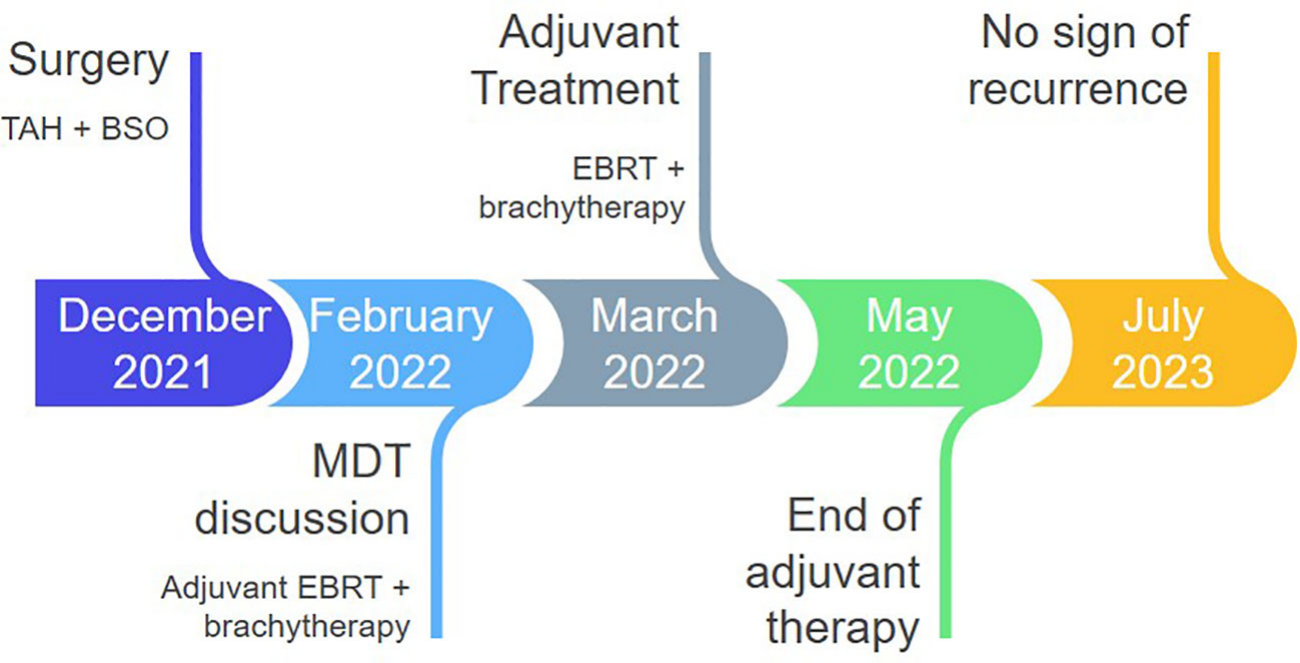
Timeline of postoperative management. TAH, total abdominal hysterectomy; BSO, bilateral salpingo-oophorectomy; MDT, multidisciplinary team board; EBRT, external beam radiotherapy.

A 45-year-old patient with genetically proven TS (heterozygous for the R1743Q mutation in exon 40 of *TSC2*) exhibiting recurrent epileptic seizures along with a history of previous left nephrectomy due to angiomyolipoma was admitted due to vaginal bleeding. Abdominal ultrasound revealed multiple hypoechoic nodules on the right kidney consistent with angiomyolipoma. Abdominal computed tomography (CT) showed enlargement of the uterus and a 3.7 cm large tumor in contact with the left parametrium. A moderately differentiated endometrioid carcinoma was diagnosed in an endometrial curettage specimen, following which the patient underwent total abdominal hysterectomy with bilateral salpingo-oöphorectomy. A solid, gray-white, 6.5 cm large lesion within the uterine corpus was found on gross examination, with two smaller, 1 cm and 0.4 cm large nodules abutting to it. Microscopic examination of the lesion revealed a FIGO grade 2 endometrioid adenocarcinoma (**Figures 1A, B**) invading the left parametrium. The carcinomatous cells exhibited strong diffuse immunopositivity for CKAE1/AE3 (**Figure 1C**), Estrogen receptor (ER, **Figure 1D**), and Progesterone receptor (PR, **Figure 1E**). Immediately abutting the carcinoma was a mesenchymal neoplasm consisting mainly (around 90% of the tumor volume) of epithelioid (**Figures 1A, F**) and focally (an estimated 10% of the tumor volume) spindle (**Figures 1A, I**) cells arranged in sheets or nests, with a clear or eosinophilic cytoplasm, lacking significant cytological atypia or nuclear pleomorphism; thin, delicate vessels were found in between the tumor cells. The mitotic count was up to 1/50 High Power Field (HPF, x400 magnification). No necrosis or lymphovascular invasion was documented. The epithelioid cells were immunostained for melanocytic glycoprotein 100 [Human Melanoma Black-45 (HMB-45), **Figure 1G**] and Microphthalmia Transcription Factor (MiTF, **Figure 1H**), as well as focally for Melan-A, whereas Desmin decorated spindle cells (**Figure 1J**). SMA (**Figure 1K**), h-caldesmon, ER, and PR stains were positive in both epithelioid and spindle cells. A diagnosis of perivascular epithelioid cell tumor (PEComa) was therefore made. Both smaller nodules were consistent with leiomyomas (**Figure 1L**). Detailed immunohistochemical results are displayed in **Table 1**. A pathological stage pT3a was assigned for the endometrial carcinoma. The postoperative course was uneventful. The patient was discharged at the 6^th^ postoperative day. The multidisciplinary tumor board opted for adjuvant radiation therapy, brachytherapy for the endometrioid carcinoma, and long-term follow-up for the PEComa. Patient underwent external beam radiotherapy with a total dose of 5000 cGy and vaginal brachytherapy as a boost after EBRT. The patient is alive 19 months postoperatively, with no sign of recurrence (normal range of CA-125, no signs on ultrasound examination and CT scan of recurrence). The timeline with relevant data from the episode of care is displayed in **Figure 2**.

**Table 1:** Cumulative data presenting immunohistochemical findings.

	PEComa Spindle-cell	PEComa Epithelioid-cell	Endometrioid carcinoma	Leiomyoma
**CKAE1/AE3**	**-**	**-**	**+**	**-**
**ER**	**+**	**+**	**+**	**+**
**PR**	**+**	**+**	**+**	**+**
**P16^INK4A^**	**F+**	**F+**	**F+**	**-**
**P53**	**-**	**-**	**-**	**-**
**MiTF**	**-**	**+**	**-**	**-**
**HMB45**	**-**	**+**	**-**	**-**
**Melan-A**	**-**	**F+**	**-**	**-**
**SMA**	**+**	**-**	**-**	**+**
**Desmin**	**+**	**-**	**-**	**+**
**Calretinin**	**-**	**-**	**-**	**-**
**h-caldesmon**	**+**	**-**	**-**	**+**
**CD117(c-kit)**	**-**	**-**	**-**	**-**
**S100**	**-**	**-**	**-**	**-**

ER, Estrogen receptor; HMB45, human melanoma black 45; Melan A, Melanoma Antigen; MiTF, Microphthalmia Transcription Factor; PR, Progesterone receptor; SMA, smooth muscle actin; F+, focal immunopositivity; D+, diffuse immunopositivity.

## Discussion

PEComas of the female genital tract present in patients in their fifth to sixth decades, usually with abnormal uterine bleeding and/or abdominal pain, although unusual presentations have been reported (7). These are extremely rare mesenchymal neoplasms, with approximately 100 cases reported, most frequently in the uterus (6, 7). PEComas are highly associated with tuberous sclerosis (TS), a rare autosomal dominant disease linked with mutations in either *TSC1* or *TSC2* genes, that map to chromosome loci 9q34 and 16p13.3 respectively (14, 15). *TSC1* and *TSC2* encode for hamartin and tuberin, respectively; both proteins form a complex which negatively regulates cell cycle progression through inhibition of mTOR (mammalian target of rapamycin) signaling (16). Inactivating mutations in either gene results in identical clinical manifestations. Our case was heterozygous for the R1743Q mutation in exon 40 of *TSC2*. Patients with TS harbor more frequent mutations in *TSC2* than *TSC1*, with exons 37 and 38 having a higher mutation frequency, whereas exon 40 is less frequently mutated (17). Translocations of transcription factor E3 (TFE3), a member of the Microphthalmia Transcription Factor (*MiTF*) gene family located in the short arm of chromosome X, have also been detected in PEComas (18, 19). Besides, missense and silent *TP53* mutations have been found in epithelioid PEComas, which may be associated with a more aggressive behavior (20); moreover, *RAD51B* fusions were detected in tumors displaying brisk mitotic activity and aggressive clinical behavior (6, 21).

Patients with TS exhibit tumors affecting multiple organs, including periungual fibromas, cardiac rhabdomyomas, giant-cell astrocytomas, chordomas, and PEComas. The most common renal manifestation is PEComa, including angiomyolipoma. Additionally, many patients exhibit neural manifestations like seizures and cognitive disorders. Non-neoplastic manifestations are also seen, such as cutaneous shagreen patches (22).

Histologically, PEComas may display pushing or invasive borders. Tumor cells can be epithelioid and/or spindled, with clear to eosinophilic granular cytoplasm (6, 23). There is variable nuclear atypia and mitotic count; tumor necrosis or lymphovascular invasion may be observed. Tumor vasculature may exhibit various forms, such as thin and delicate vessels, thin and ectatic vessels, thick-walled vessels, or hemangiopericytomatous-like vessels (7, 23). Uterine PEComas may rarely show morphological features akin to lymphangioleiomyomatosis (6, 24).

Immunohistochemically, PEComas express myomelanocytic markers with variable extent and intensity of staining. HMB-45, Melan-A and MiTF are variably expressed, often displaying only minimal staining (7). Smooth muscle markers tend to stain diffusely and strongly in spindle cells, whereas melanocytic markers are likewise expressed in epithelioid cells (25). Cathepsin K is often expressed strongly and diffusely, while PNL2, a novel melanocytic marker, displays variable cytoplasmic immunostaining among PEComas (26, 27).

Preoperative diagnosis of these tumors is problematic because a wide range of tumors, including leiomyoma, low-grade endometrial stromal sarcoma, uterine tumor resembling ovarian sex cord tumors (UTROSCT), other metastatic sarcomas such as a gastrointestinal stromal tumor, as well as melanoma, are included in the differential diagnosis. Besides, since this entity is very rare, its diagnosis is challenging. Leiomyomas may cause the most challenging differential diagnostic problems because they show morphologic features similar to PEComas. The presence of perinuclear vacuoles, diffuse eosinophilic cytoplasm without granularity, thick-walled vessels, and lack of thin and delicate vasculature characteristic of PEComas may provide helpful diagnostic clues. Immunohistochemically, leiomyomas may focally decorate for both melanocytic markers and Cathepsin K (7). Low-grade endometrial stromal sarcoma morphology is similar to proliferative phase endometrium. Gastrointestinal stromal tumors may also resemble PEComas, in which the expression of CD34, CD117, and DOG-1 is diagnostically useful. UTROSCTs display a morphological variability, showing small nests, broad trabeculae, anastomosing cords, glomeruloid or cystic structures, retiform or sertoliform tubules, and prominent Leydig-like cells. Immunohistochemically they may express markers of sex cord (calretinin, inhibin, and CD99), smooth muscle differentiation (SMA, desmin, and caldesmon) and melanocytic (Melan-A) differentiaton. In addition, they may sometimes express epithelial markers or CD10 (28). Melanoma may show different growth patterns, with highly pleomorphic cells displaying macronucleoli; nevertheless, melanoma cells do not express myogenic markers (7).

Three different diagnostic algorithms have been proposed to assess the biological potential of PEComas. Based on modified gynecology-specific criteria (6), soft tissue and gynecologic PEComas are classified into three categories, namely benign, of uncertain malignant potential, and malignant, based on size, type of tumor front (pushing or infiltrative), nuclear grade, mitotic count, necrosis, and vascular invasion (18). The other two approaches classify PEComas of the female genital tract into uncertain malignant potential and malignant classes based on the assessment of gross size, nuclear features, necrosis, vascular invasion, and mitotic rate with the algorithm proposed by Schoolmeester et al. (23) requiring four criteria to classify as malignant. In contrast, the algorithm proposed by Bennett et al. (6) requires three or more criteria to qualify for malignancy. Our case is stratified as uncertain malignant potential according to modified gynecology-specific criteria.

Molecular studies have recognized four distinct subtypes of endometrial carcinomas: copy number-high, copy number-low, microsatellite instability hypermutated and polymerase ϵ (POLE) ultramutated (29, 30). Also nuclear expression of β-catenin is usually related with CTNNB1 mutations that are associated with an adverse prognosis in patients with low grade, low risk endometrial carcinoma (31). This classification assists in patient risk stratification and management. Immunohistochemical staining on formalin-fixed paraffin embedded tissue blocks for mismatch repair system proteins (MMR) p53 and β-catenin can be used as surrogate markers and aid in molecular classification (32, 33). In our case nuclear expression of all four MMR (MLH1+/MSH2+/PMS2+/MSH6+) on immunohistochemical evaluation of both tumor components (carcinoma and PEComa) was retained. Besides, the carcinoma expressed cytoplasmic β-catenin as well as focal/faint p53 which is considered negative.

We found two previously reported similar cases with the coexistence of uterine PEComa, endometrioid carcinoma, and uterine leiomyoma (34, 35) (**Table 2**). Case 1 (Choi et al.) concerned a malignant PEComa, whereas in case 2 (Gao et al.), the PEComa had benign features. To our knowledge, ours is the first case reported in a patient with genetically proven tuberous sclerosis.

**Table 2:** Clinical and histopathological characteristics of two cases with synchronous uterine endometrial carcinoma and PEcoma along with leiomyoma.

	Case 1	Case 2
**Age (years)**	67	60
**Initial presentation**	Vaginal Bleeding	Vaginal bleeding
**TSC background**	ND	No
**Uterine endometrioid carcinoma – tumor size (maximum diameter, cm)**	4	2
**Uterine endometrioid carcinoma – microscopic findings**	Invasion of more than half of the myometrium – Well differentiated endometrial carcinoma	Myometrium invasion with extensive necrosis - Well-differentiated endometrial carcinoma
**PEcoma – tumor size (maximum diameter, cm)**	6	4
**PEcoma – morphological findings**	Spindle to ovoid cells with clear cytoplasm, atypia, and prominent nucleoli, presence of coagulative necrosis, mitotic rate 14/50 HPF,	Epithelioid and spindle cells with mild to moderate nuclear polymorphism, absence of necrosis, and no mitotic figures
**PEcoma – immunostaining**	IHC Panel: HMB-45, Melan-A, CD10, a-SMA, caldesmon, desmin, TFE3, Ki67 Tumor cells: HMB-45(+), SMA(+), TFE3(+), Ki-67(<5%)	IHC Panel: HMB-45, Melan-A, CD10, CD68, SMA, caldesmon, desmin, Cytokeratin, Vimentin, CD117, S-100 Epithelioid cells: HMB-45(+, focal), Melan-A (+, diffuse and strong), CD10(+, focal and weak), CD68(+, diffuse and strong); SMA(+), Caldesmon(+), Desmin(+) Spindle cells: SMA(+), Caldesmon(+), Desmin(+)
**Other**	Leiomyoma, multiple liver, and lung metastasis	Leiomyomata, Serous cystadenofibroma (left ovary)
**Reference**	Choi et al., 2016 (34)	Gao et al., 2004 (35)

HPF, High power field (magnification 400x); IHC, Immunohistochemistry; ND, not determined; TSC, Tuberous sclerosis.

Currently, surgery constitutes the treatment of choice for PEComas, with hysterectomy (with or without bilateral salpingo-oöphorectomy); a long-term follow-up is mandatory due to the possibility of local recurrence or metastasis, usually to the lung, even several years post-operation (9, 19, 21, 36–38). Cases with oligometastatic disease are handled with surgery when feasible. Systemic chemotherapy has so far shown poor results (39), in contrast to targeted therapies, especially with mTOR and VEGFR inhibitors, which have been promising (38–40). The role of adjuvant radiotherapy has not been determined (39). Given the small number of cases, the optimal therapeutic approach for uterine PEComas is debated.

## Conclusions

In summary, we hereby report a collision tumor composed of PEComa, endometrioid endometrial carcinoma, and two leiomyomas in a patient with genetically proven tuberous sclerosis, the first case reported, to our best knowledge. Furthermore, we review the relevant literature focusing on the differential diagnostic issues and the diagnostic criteria of malignancy and discuss the molecular findings and treatment options for PEComas.

## Data availability statement

The original contributions presented in the study are included in the article/supplementary material. Further inquiries can be directed to the corresponding author.

## Ethics statement

The studies involving human participants were approved by the Attikon Hospital Ethics Committee (EΣ121/28-03-2023) and conducted in accordance with the 1964 Helsinki Declaration, and its later amendments, and the local legislation and institutional requirements. The participants provided their written informed consent to participate in this study. Written informed consent was obtained from the participant/patient(s) for the publication of this case report.

## Author contributions

Conceptualization: NK; ISP; Data curation: CK; A-RG; Formal analysis: A-II; AF; Funding acquisition: NV; Investigation: A-RG.; Methodology: A-RG; A-II; Project administration: IGP; NV Resources: CK; A-II.; Software: CK; A-RG; Supervision: IGP; NV; Validation: AF; CK; Visualization: AF; A-RG; Writing - original draft: NK; ISP; Writing - review & editing: NK; ISP; CK; A-RG; A-II; AF; IGP; NV.
